# Evaluation of Right Heart Structure and Function in Pacemaker-dependent Patients by Two-Dimensional Speckle Tracking Echocardiography: A 1-Year Prospective Cohort Study

**DOI:** 10.31083/j.rcm2511408

**Published:** 2024-11-20

**Authors:** Yingchen Mei, Rui Han, Liting Cheng, Haiwei Li, Yihua He, Wei Liu, Yongquan Wu

**Affiliations:** ^1^Department of Cardiology, Beijing Jishuitan Hospital, Capital Medical University, 100035 Beijing, China; ^2^Cardiac Pacing and CIED Center, Beijing Anzhen Hospital, Capital Medical University, 100029 Beijing, China; ^3^School of Medicine, Nankai University, 300071 Tianjin, China; ^4^Echocardiography Medical Center, Beijing Anzhen Hospital, Capital Medical University, 100029 Beijing, China

**Keywords:** left bundle branch area pacing, two-dimensional speckle tracking echocardiography, right ventricular, interventricular synchrony, tricuspid regurgitation

## Abstract

**Background:**

Left bundle branch area pacing (LBBAP) has evolved into a practical and secure pacing procedure. However, previous studies of LBBAP focused on left heart function and synchronization and lacked assessment of right heart structure and function and interventricular synchrony. The objective of this study was to examine the impacts of LBBAP, right ventricular (RV) septal pacing (RVSP), and RV apical pacing (RVAP) on right heart structure, function and interventricular synchrony.

**Methods:**

Between January and July 2021, A total of 90 patients exhibited a normal left ventricular (LV) ejection fraction and received dual chamber pacemaker implantation for bradycardia at Beijing Anzhen Hospital. The patients were assigned to three groups based on the pacing site: LBBAP, RVSP, or RVAP. RV function was evaluated using right ventricular fractional area change (RVFAC), tricuspid annular plane systolic excursion (TAPSE), tissue Doppler–derived tricuspid lateral annular systolic velocity (S'), right ventricular myocardial performance index (RVMPI), global longitudinal strain of the right ventricle (GLSRV), and right ventricular free wall longitudinal strain (RVFWLS). Tricuspid regurgitation (TR) was assessed using vena contracta magnitude (VCM) and the ratio of TR jet area to right atrial area (RAA). Interventricular mechanical synchrony was evaluated using interventricular mechanical delay (IVMD) and left ventricular to right ventricular time-to-peak standard deviation (LV-RV TPSD).

**Results:**

Baseline echocardiographic parameters and characteristics were comparable among the three groups. No significant differences were observed in the LBBAP group from baseline to follow-up for QRS duration (*p* = 0.783), TAPSE (*p* = 0.122), RVFAC (*p* = 0.679), RVMPI (*p* = 0.93), GLSRV (*p* = 0.511), RVFWLS (*p* = 0.939), VCM (*p* = 0.467), and TR jet area/RAA (*p* = 0.667). In contrast, a significant decline was observed in the RVAP group (all *p* < 0.05). RVSP resulted in a similar percentage reduction in TAPSE, GLSRV, and RVFWLS (all *p* > 0.05). However, there were significant differences in RVFAC (*p* = 0.009), RVMPI (*p* = 0.037), TRVCM (*p* = 0.046), and TR jet area/RAA (*p* = 0.033) in the RVSP group. Moreover, compared to baseline, a 1-year follow-up showed that LBBAP significantly reduced IVMD (from 17.3 ± 26.5 ms to 8.6 ± 7.1 ms, *p* < 0.05) and LV-RV TPSD [from 16.41 (8.81–42.5) to 12.28 (5.64–23.7), *p* < 0.05], while RVSP and RVAP worsened IVMD and LV-RV TPSD (all *p* < 0.05).

**Conclusions:**

Compared with RVSP or RVAP, LBBAP can maintain RV function and improve electrical and interventricular synchrony, with limited TR deterioration after a 1-year follow-up.

**Clinical Trial Registration:**

No. ChiCTR2100048503, https://www.chictr.org.cn/showproj.html?proj=129290.

## 1. Introduction

Pacemaker implantation is commonly used to manage symptomatic bradycardia and cardiac conduction disorders. Conventional right ventricular (RV) pacing, on the other hand, has the potential to cause dysynchrony between the ventricles and the left side, which increases the risk of decreased left ventricular (LV) function. According to certain reports, impaired RV function may also result from altered RV activity [1]. Left bundle branch (LBB) area pacing (LBBAP) is a feasible and secure alternative to RV pacing (RVP) that produces a narrower QRS interval, a sustained low pacing output, and improved mechanical synchrony [2, 3]. Nevertheless, the observed QRS morphology in cases of LBBAP indicates the presence of the right bundle branch block (RBBB) in lead V1, suggesting that the order of agitation of the LV takes precedence over the RV. Previous studies of LBBAP focused on left and lacked assessment of right heart structure and function and interventricular synchrony [4, 5].

Although parameters measured via two-dimensional echocardiography, such as the tricuspid annular plane systolic excursion (TAPSE), RV fractional area change (RV FAC), tissue Doppler–derived tricuspid lateral annular systolic velocity (S’), and RV myocardial performance index (MPI), are frequently applied for the assessment of RV function, the complex geometry of the right heart complicates evaluation of right atrial (RA) and RV structure and function through conventional echocardiographic parameters [6, 7]. Instead, two-dimensional speckle tracking echocardiography (2D-STE) has been proposed as a viable method for assessing RA function as well as global and regional myocardial function performance of the RV [8].

Because the impacts of LBBAP on the performance of the RA and RV have not been thoroughly investigated, the objective of the present investigation was to assess and compare the anatomical and physiological attributes of the right heart over 1 year after treatment with LBBAP, RV septal pacing (RVSP), or RV apical pacing (RVAP) using conventional echocardiography in conjunction with 2D-STE.

## 2. Materials and Methods

### 2.1 Study Population and Ethical Considerations

From January 2021 to July 2021, a cohort of consecutive patients who met the criteria for elective permanent dual chamber pacemaker implantation, as outlined in the current recommendations (Class I) [9], were prospectively enrolled at the Beijing Anzhen Hospital. The inclusion criteria were: (1) age >18 years; (2) diagnosis of sick sinus syndrome (dual nodal lesions) and persistent high-grade atrioventricular block, with ventricular pacing ratio exceeding 70%; and (3) left ventricular ejection fraction (LVEF) >50% at baseline. Patients who met any of the following exclusion criteria were not included: presence of moderate to severe valvular disease; New York Heart Association (NYHA) functional class III or IV; history of acute and chronic coronary syndrome requiring intervention or revascularization within the previous 3 months; documented medical history of cardiomyopathy or tachyarrhythmia such as persistent atrial fibrillation; diagnosis of chronic obstructive pulmonary disease, liver failure, or kidney failure; current pregnancy; poor quality of ultrasound images; or inability to adhere to regular clinic follow-up appointments.

The included participants were assigned to either the LBBAP, RVSP, or RVAP group based on the site of pacing.

The research protocol was approved by the Beijing Anzhen Hospital Medical Ethics Committee (No. 2021083X) and was consistent with the ethical principles delineated in the 1975 Declaration of Helsinki. Patients provided written informed consent before study participation. The study was registered with the Chinese Clinical Trial Registry (No. ChiCTR2100048503).

### 2.2 Pacemaker and Lead Implantation

As previously described [10], the LBBAP procedure was conducted with the Select Secure pacing lead (model 3830, 69 cm) delivered through the C315 fixed rigid curved sheath (Medtronic, Minneapolis, MN, USA). The LBB was initially assessed using a right anterior oblique 30^∘^ fluoroscopic view. The appropriate pacing area was selected using the New Nine Partition Method [11]. The 3830 lead and C315 sheath were directed toward the interventricular septum and collectively spun in a clockwise direction. The lead was affixed by executing 8–10 clockwise rotations during the process of pace mapping. This was done after a W-shaped QRS morphology in V1 was seen at the tip of the lead. The lead was considered to have resolved when the QRS morphology in V1 exhibited a “QR/Qr” pattern, and the time interval from the pacing stimulus (Stim) to LV activation time (LVAT) in V5 or V6 was less than 75 ms and remained consistent regardless of high or low outputs. Threshold testing was conducted during the process of implantation using a Medtronic CareLink™ Programmer (Minneapolis, MN, USA). LBBAP was considered successful when all three of the following criteria were met, as stated by Zhang *et al*. [12]: (1) the final location of the 3830 lead, as determined by fluoroscopic imaging, was in the vicinity of the LBB; (2) unipolar-paced morphology exhibited a narrow QRS (<130 ms) and an RBBB pattern; and (3) the time from Stim to peak LVAT in V4–V6 was <90 ms and remained consistently brief at both low and high outputs, indicating that the LBB was captured. Further indications of effective LBBAP were provided by direct LBB capture evidence, which included the LBB potential, selective LBBAP, and nonselective LBBAP detected by an electrophysiological recording device.

The RV leads were placed at either the RV apex or septum following standard procedure.

### 2.3 Electrocardiography

A 12-lead surface electrocardiogram (ECG) was performed at 100 mm/s with a GE Cardiolab Electrophysiology recording system (GE Healthcare Inc, Marlborough, MA, USA) before and after implantation. An intracardiac electrogram (IEGM) was obtained during implantation from the tip electrode of the 3830 lead. The intrinsic QRS duration (QRSd), paced QRSd, and Stim to LVAT time were measured sequentially. QRSd was calculated as the time from the onset to the end of the intrinsic or paced QRS complex in all 12 leads of the ECG. The Stim to LVAT time was measured as the interval from stimulus to the peak of the R wave in V4–V6.

### 2.4 Echocardiography

Transthoracic two-dimensional (2D) echocardiography was conducted using S5-1 transducers and the EPIQ 7C ultrasound system (Philips, Bothell, WA, USA). The modified apical four-chamber view was utilized to acquire images of the RA and RV, with careful attention given to capturing the full structures of both. Current guidelines were applied to grade the severity of tricuspid regurgitation (TR), based on the vena contract width, proximal flow convergence, and regurgitant jet [13].

Measurement of the RV dimensions at the base during end diastole was conducted using an RV-focused apical 4-chamber view. RV systolic function is typically estimated by measuring the FAC, TAPSE, and the systolic velocity S-wave of the tricuspid annulus on tissue Doppler imaging (TDI). The calculation of the MPI, which represents a comprehensive measure of both systolic and diastolic RV performance, involves dividing the isovolumetric time intervals by the ventricular ejection time. The interventricular mechanical delay (IVMD) was employed to assess the mechanical synchronization between the two ventricles, as determined by pulse wave Doppler imaging. These parameters were obtained following the latest recommendations [14].

### 2.5 Speckle Tracking Echocardiography 

Analysis of 2D-STE data was conducted on 2D images of the RV-focused four-chamber view. The data were analyzed using the specialized software QLAB V.13.0 developed by Philips Medical Systems (Bothell, WA, USA). The data were acquired from four consecutive cardiac cycles. Concurrently with the cardiac cycles, an ECG was acquired to determine the heart rate. RA strain was applied during the reservoir phase (RASr), the conduit phase (RAScd), and the contraction phase (RASct). The RV strain parameters of RV free wall longitudinal strain (RVFWLS) and global longitudinal strain of the RV (GLSRV) were acquired according to the most recent recommendations [8]. Dysynchrony was evaluated as time-to-peak standard deviation (TPSD), and we also evaluated interventricular dysynchrony, based on left ventricular to right ventricular TPSD (LV-RV TPSD) using the same RR interval (The RR interval refers to the time interval or period between two consecutive R-waves in a cardiac cycle).

### 2.6 Data Collection and Follow-Up

Patients’ clinical and demographic characteristics were recorded at baseline. During the stages of implantation, predischarge, and 1-year follow-up, data regarding the device were collected. Identical equipment was utilized for both the baseline and follow-up examinations. Two seasoned and impartial specialists, who were also blinded to the research, examined and analyzed the parameters.

### 2.7 Statistical Analysis

The Shapiro-Wilk test was utilized to assess data normality. Continuous variables are presented as mean and standard deviation (SD) or median and interquartile range (IQR), whereas categorical variables are presented as frequency and percentage. Continuous variables were compared using the independent-samples *t*-test, and categorical variables were compared using the χ^2^ test. Data were compared among the three subgroups by one-way analysis of variance. Changes in variables from before to after follow-up in the same group were identified by paired *t*-test. Differences for which *p* < 0.05 were deemed to be statistically significant. All statistical analyses were conducted using SPSS Statistics V22.0 (IBM Corp, Armonk, NY, USA).

## 3. Results

### 3.1 Patient Characteristics

**Fig. 1.**
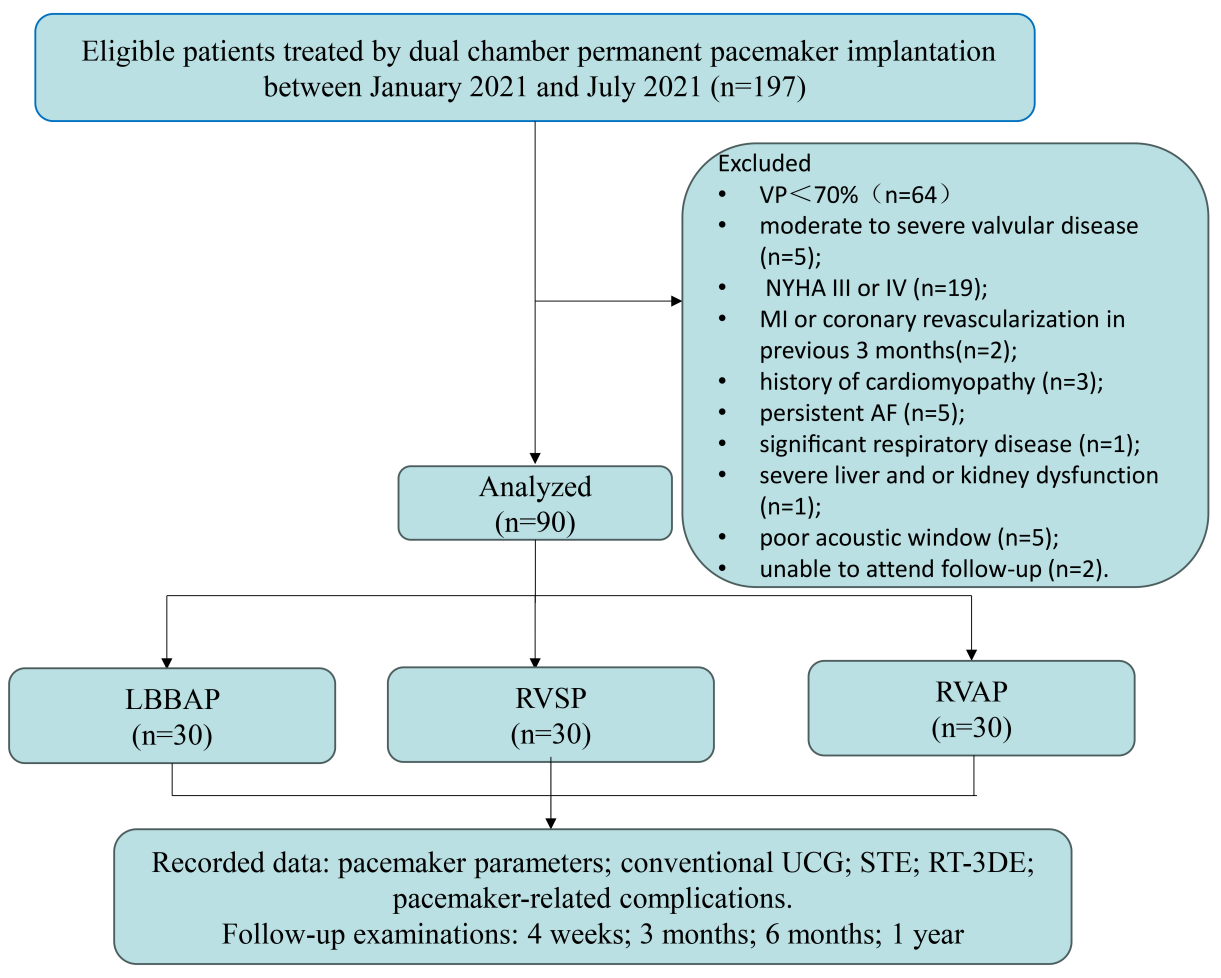
**Flow chart of the prospective cohort study**. Abbreviations: UCG, ultrasound cardiography; STE, speckle tracking echocardiography; RT-3DE, real-time three-dimensional echocardiography; NYHA, New York Heart Association; MI, myocardial infarction; AF, atrial fibrillation; VP, ventricular pacing; LBBAP, left bundle branch area pacing; RVSP, right ventricular septal pacing; RVAP, right ventricular apical pacing.

A total of 197 consecutive patients underwent elective permanent dual-chamber pacemaker implantation during the study period. 97 were included in the study population after application of the inclusion and exclusion criteria, as outlined in Fig. 1. Another seven patients were then excluded from the final analysis due to unsatisfactory 2D-STE tracking data. One patient in the LBBAP group (1/31, 3.23% of total sample size) experienced an unsatisfactory outcome and was subsequently treated with RVSP. For the final analysis, each group included 30 patients.

Table 1 displays the demographic and clinical characteristics of the final study population. No statistically significant differences were observed in baseline characteristics, clinical disease diagnoses, underlying comorbidities, or medication use among the three groups.

**Table 1.** **Demographic and clinical characteristics of the patient population by treatment group**.

Variables		LBBAP (n = 30)	RVSP (n = 30)	RVAP (n = 30)	F/χ^2^	*p*
Age (years)		62.45 ± 13.29	66.15 ± 12.27	63.41 ± 15.27	0.543	0.583
Male gender		18 (60%)	15 (50%)	16 (53.3%)	0.376	0.829
BMI (kg/m^2^)		25.74 ± 5.22	25.85 ± 2.68	24.64 ± 3.36	0.815	0.446
Systolic blood pressure (mm Hg)		135.26 ± 14.94	135.96 ± 16.37	136.24 ± 16.01	0.031	0.969
Diastolic blood pressure (mm Hg)		74.45 ± 8.28	75.31 ± 11.19	74.03 ± 9.11	0.127	0.881
Resting heart rate (beats/min)		55.77 ± 11.45	60.58 ± 19.89	58.28 ± 10.42	0.807	0.45
Comorbidities						
	Hypertension	17 (56.67%)	17 (56.67%)	15 (50%)	1.134	0.567
	Coronary heart disease	3 (10%)	4 (13.33%)	3 (10%)	2.113	0.576
	Diabetes mellitus	10 (33.33%)	9 (30%)	7 (23.33%)	2.377	0.468
	Stroke	1 (3.33%)	3 (10%)	2 (6.68%)	1.506	0.471
	Chronic kidney disease	1 (3.33%)	1 (3.33%)	0 (0%)	1.066	0.587
	Hyperlipidemia	6 (20%)	8 (26.67%)	9 (30%)	1.351	0.509
	Pulmonary hypertension	0	0	0		
Clinical diagnosis						
	Sick sinus syndrome	6 (20%)	5 (16.67%)	6 (20%)	1.153	0.667
	Atrioventricular block	24 (80%)	25 (83.33%)	24 (80%)	2.056	0.559
	LBBB	3 (10%)	3 (10%)	2 (6.68%)	2.234	0.534
	RBBB	5 (16.67%)	4 (13.33%)	5 (16.67%)	2.014	0.425
Medications						
	Aspirin	7 (23.33%)	8 (26.67%)	4 (13.33%)	2.302	0.316
	Anticoagulant drugs	5 (16.67%)	6 (20%)	4 (13.33%)	2.123	0.435
	Ace-inhibitor/ARB	9 (30%)	15 (50%)	10 (33.33%)	5.326	0.07
	Beta-blocker	1 (3.33%)	1 (3.33%)	1 (3.33%)	0.016	0.992
	Calcium-antagonists	10 (33.33%)	12 (40%)	11 (36.66%)	1.229	0.678
	Insulin treatment	7 (23.33%)	7 (23.33%)	4 (13.33%)	1.231	0.459
	Statin use	8 (26.67%)	11 (36.67%)	7 (23.33%)	2.596	0.273
	Smoking history	10 (33.33%)	6 (20%)	10 (33.33%)	0.94	0.625
	Alcohol consumption	3 (10%)	2 (6.68%)	5 (16.68%)	1.348	0.498
ECG (ms)						
	Pre-pacing PR interval	206.65 ± 70.82	200.77 ± 66.87	195.17 ± 55.2	0.236	0.791
	Pre-pacing QRS duration	111.26 ± 24.07	102.88 ± 18.78	101.55 ± 16.09	2.057	0.134
	Post-pacing QRS duration	114.45 ± 26.23^*^	133.5 ± 28.34^‡^	157.29 ± 29.18^†^	4.671	0.012
	QRS duration Change	3.19 ± 3.51^*^	30.62 ± 7.66^‡^	55.74 ± 9.94^†^	7.983	0.001
	*p*	0.783	0.001	0.001		

Abbreviations: BMI, body mass index; LBBB, left bundle branch block; RBBB, right bundle branch block; ARB, angiotensin receptor blocker; LBBAP, left bundle branch area pacing; RVSP, right ventricular septal pacing; RVAP, right ventricular apical pacing; ECG, electrocardiogram. Post-pacing QRS duration was measured from stimulus to the end of the last QRS complex deflection in the 12-lead electrocardiogram; Pre-pacing QRS duration was measured from the first to last sharp vector of the QRS complex crossing the isoelectric line 12-lead electrocardiogram. ^†^*p* < 0.05 RVAP vs. LBBAP, ^*^p < 0.05 LBBAP vs. RVSP, ^‡^*p* < 0.05 RVSP vs. RVAP.

### 3.2 RV Function 

The echocardiographic parameters for patients in each group are detailed in Table 2. No significant differences were detected among the three groups at baseline. At the 1-year follow-up, we observed that the LBBAP group could maintain right heart function, based on differences in RV FAC (50.81% ± 7.57% with LBBAP vs. 45.43% ± 6.91% with RVSP vs. 44.29% ± 7.98% with RVAP, *p* = 0.006), TAPSE (25.37 ± 4.7 mm vs. 22.83 ± 4.03 mm vs. 22.66 ± 3.96 mm, *p* = 0.017); and RVMPI (0.31% ± 0.15% vs. 0.37% ± 0.24% vs. 0.55% ± 0.33%, *p* = 0.015), while the RVSP and RVAP groups showed different degrees of impairment in right heart function. In addition to this, we found that no significant difference was seen in the change from baseline to follow-up in the LBBAP group for the following parameters: RVFAC (49.31 ± 7.6% with baseline vs. 50.81 ± 7.57% with follow-up, *p* = 0.679); TAPSE (23.43 ± 4.6 mm vs. 25.37 ± 4.7 mm, *p* = 0.122); RV MPI (0.45 ± 0.18% vs. 0.31 ± 0.15%, *p* = 0.93). RVSP resulted in a similar percentage reduction in TAPSE (*p* > 0.05). In contrast, significant differences were observed in RVFAC (*p* = 0.09) and RVMPI (*p *= 0.037). RVSP resulted in a similar percentage reduction in TAPSE (*p* > 0.05), while there were significant statistical differences in RVFAC (*p* = 0.009), RVMPI (*p* = 0.037). RVFAC, TAPSE, and RVMPI significantly decreased in the RVAP group. This indicates that LBBAP does not impair RV function in contrast to RVSP and RVAP.

**Table 2.** **Conventional echocardiographic measurements for patients in the three groups**.

Variables			LBBAP (n= 30)	RVSP (n = 30)	RVAP (n = 30)	F/χ^2^	*p*
RV basal diameter (mm)							
	Baseline		38.07 ± 4.46	35.73 ± 4.47	35.33 ± 6.31	2.401	0.097
	One-year follow-up		37.5 ± 4.61	36.08 ± 4.74	36.35 ± 5.34	0.599	0.552
	Change		0.19 ± 2.15	0.16 ± 3.33	1.08 ± 3.89	1.078	0.346
	*p*		0.653	0.809	0.171		
RV mid diameter (mm)							
	Baseline		28.8 ± 5.57	26.96 ± 4.46	25.63 ± 5.19	2.888	0.061
	One-year follow-up		27.08 ± 4.34	27.21 ± 3.39	26.96 ± 4.65	0.022	0.979
	Change		1.72 ± 3.51	0.37 ± 2.76	1.38 ± 3.42	2.85	0.065
	*p*		0.273	0.513	0.049		
RV longitudinal diameter (mm)							
	Baseline		60.0 ± 6.8	62.1 ± 7.1	61.5 ± 7.2	0.987	0.346
	One-year follow-up		59.22 ± 6.51	63.1 ± 7.2	61.8 ± 7.3	0.476	0.577
	Change		0.78 ± 2.62	0.11 ± 4.91	0.30 ± 2.87	0.550	0.579
	*p*		0.941	0.437	0.113		
RVOT (mm)							
	Baseline		28.87 ± 3.41	28.42 ± 3.02	28.02 ± 4.15	0.268	0.766
	One-year follow-up		28.65 ± 3.85	28.5 ± 3.09	28.38 ± 3.91	0.036	0.965
	Change		0.22 ± 1.74	0.21 ± 1.35	0.15 ± 1.64	0.474	0.624
	*p*		0.579	0.458	0.637		
RV FAC (%)							
	Baseline		49.31 ± 7.6	49.58 ± 7.48	52.7 ± 6.86	1.131	0.148
	One-year follow-up		50.81 ± 7.57^*^	45.43 ± 6.91^‡^	44.29 ± 7.98^†^	5.542	0.006
	Change		1.5 ± 7.48^*^	4.15 ± 8.73^‡^	8.41 ± 10.8^†^	7.082	0.002
	*p*		0.679	0.009	0.001		
TAPSE (mm)							
	Baseline		23.43 ± 4.60	23.28 ± 4.38	24.94 ± 5.82	0.976	0.381
	One-year follow-up		25.37 ± 4.7	22.83 ± 4.03	22.66 ± 3.96	2.654	0.017
	Change		2.11 ± 6.78	0.71 ± 4.62	3.16 ± 5.73^†^	5.478	0.006
	*p*		0.122	0.94	0.09		
S’ (cm/sec)							
	Baseline		16.94 ± 4.25	13.54 ± 4.19	14.3 ± 4.08	0.796	0.454
	One-year follow-up		17.48 ± 5.17	14.3 ± 4.08	11.85 ± 2.53	1.343	0.294
	Change		0.59 ± 3.57	0.81 ± 3.15	2.45 ± 1.79	1.84	0.166
	*p*		0.121	0.473	0.001		
Tissue Doppler MPI							
	Baseline		0.45 ± 0.18	0.48 ± 0.13	0.48 ± 0.12	0.551	0.578
	One-year follow-up		0.31 ± 0.15^*^	0.37 ± 0.24^‡^	0.55 ± 0.33^†^	5.328	0.015
	Change		0.06 ± 0.35^*^	0.11 ± 0.25^‡^	0.17 ± 0.21^†^	2.985	0.046
	*p*		0.93	0.037	0.001		
RA diameter (mm)							
	Baseline		37.58 ± 6.74	37.54 ± 6.64	37.49 ± 6.34	0.41	0.765
	One-year follow-up		38.03 ± 6.55	38.01 ± 5.98	38.45 ± 6.23	0.54	0.414
	Change		0.45 ± 6.11	0.47 ± 5.69	0.96 ± 6.23^†^	0.66	0.548
	*p*		0.157	0.167	0.231		
TR degree							
	TR VCM (cm)						
		Baseline	0.33 ± 0.23	0.33 ± 0.25	0.34 ± 0.35	1.156	0.442
		One-year follow-up	0.34 ± 0.11	0.40 ± 0.10	0.41 ± 0.12	4.183	0.035
		Change	0.01 ± 0.33	0.07 ± 0.26^‡^	0.07 ± 0.33^†^	5.167	0.017
		*p*	0.467	0.046	0.036		
	TR jet area/RAA (%)						
		Baseline	13.46 ± 1.33	12.56 ± 1.35	13.15 ± 1.34	1.114	0.557
		One-year follow-up	13.34 ± 3.39	15.44 ± 2.99	15.42 ± 3.90	3.442	0.015
		Change	0.12 ± 2.88	2.88 ± 1.98^‡^	1.92 ± 2.24^†^	5.178	0.006
		*p*	0.667	0.033	0.017		
	IVMD (ms)						
		Baseline	17.3 ± 26.5	17.6 ± 29.8	17.4 ± 28.4	0.533	0.768
		One-year fellow up	8.6 ± 7.1^*^	29.7 ± 9.9^‡^	44.7 ± 12.8^†^	5.546	0.007
		Changes	8.7 ± 5.4^*^	12.1 ± 9.7^‡^	27.3 ± 10.7^†^	3.018	0.036
		*p*	0.001	0.034	0.001		

Abbreviations: LBBAP, left bundle branch area pacing; RVSP, right ventricular septal pacing; RVAP, right ventricular apical pacing; RV,right ventricular; RVOT, right ventricular outflow tract; FAC, fractional area change; IVMD, interventricular mechanical delay; TAPSE, tricuspid annular plane systolic excursion; MPI, myocardial performance index; RA, right atrium; S’, tissue Doppler–derived tricuspid lateral annular systolic velocity TR, tricuspid regurgitation; VCM, vena contracta magnitude; RAA, right atrium area. ^†^*p* < 0.05 RVAP vs. LBBAP, ^*^p < 0.05 LBBAP vs. RVSP, ^‡^*p* < 0.05 RVSP vs. RVAP.

**Fig. 2.**
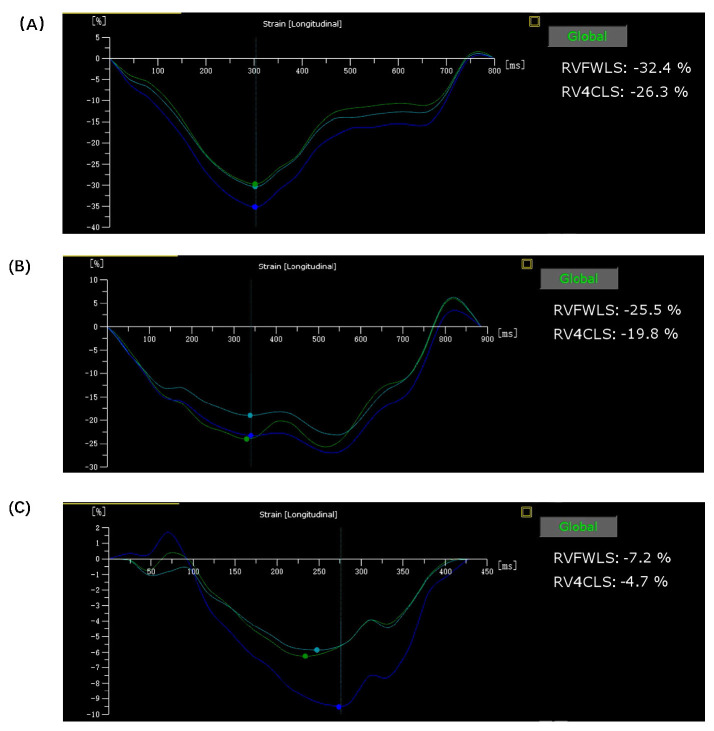
**RV strain curves after LBBAP, RVSP, or RVAP**. Representative right ventricular (RV) strain curves for patients that underwent LBBAP (A), RVSP (B), or RVAP (C). In comparison to the other two groups, the LBBAP group exhibited the most uniform and consistent curves, while the RVSP group displayed a minor degree of disorder in its curves at the 1-year follow-up. Abbreviations: RV4CLS, right ventricular four-chamber longitudinal strain; RVFWLS, RV free wall longitudinal strain; LBBAP, left bundle branch area pacing; RVSP, right ventricular septal pacing; RVAP, right ventricular apical pacing.

At the 1-year follow-up, parameters of RV strain assessed by speckle tracking echocardiography (STE), including the global longitudinal strain of the RV (GLSRV) (–21.77% ± 5.99% with LBBAP vs. –18.82% ± 5.25% with RVSP vs. –17.33% ± 3.93% with RVAP, *p* = 0.001) and RV free wall longitudinal strain (RVFWLS) (–26.43% ± 5.53% vs. –22.71% ± 4.52% vs. –22.1% ± 5.92%, *p* = 0.008), were better in the LBBAP group than in the RVSP or RVAP group. Furthermore, there were no significant changes in GLSRV (from –21.62 ± 4.28% at baseline to –21.77 ± 5.99% at follow-up, *p* = 0.511) and RVFWLS (from –26.4 ± 6.05% to –26.43 ± 5.53%, *p* = 0.939) in the LBBAP group. Similar to the results in the LBBAP group, there were no significant differences from baseline to 1 year of follow-up in either GLSRV (from –20.17 ± 5.28% to –18.82 ± 5.25%, *p* = 0.203) or RVFWLS (from –24.24 ± 7.07% to –22.71 ± 4.52%, *p* = 0.353) in the RVSP group. In contrast, data from the RVAP group at the 1-year follow-up demonstrated a notable decline in both GLSRV (from –21.95 ± 6.29% at baseline to –17.33 ± 3.93% at follow-up, *p* = 0.029) and RVFWLS (from –26.92 ± 8.18% to –22.1 ±5.92%, *p* = 0.025) (Table 3, Fig. 2).

**Table 3.** **STE of the RA and RV**.

			LBBAP (n = 30)	RVSP (n = 30)	RVAP (n = 30)	F/χ^2^	*p*
RA strain							
	RA reservoir (%)						
		Baseline	32.36 ± 2.86	34.38 ± 3.71	30.28 ± 3.219	0.384	0.683
		One-year follow-up	29.78 ± 2.58	32.54 ± 2.85	29.5 ± 1.99	0.926	0.401
		Change	2.58 ± 9.77	1.85 ± 8.72	0.78 ± 7.92	0.184	0.832
		*p*	0.412	0.589	0.938		
	RA conduit(%)						
		Baseline	–20.55 ± 2.33	–24.03 ± 2.56	–21.23 ± 2.07	0.611	0.546
		One-year follow-up	–17.58 ± 1.59	–18.81 ± 1.88	–17.68 ± 1.42	0.171	0.843
		Change	2.04 ± 4.22	5.48 ± 3.99	3.19 ± 3.83	0.438	0.647
		*p*	0.477	0.067	0.167		
	RA contraction (%)						
		Baseline	–10.43 ± 1.844	–10.35 ± 2.52	–9.11 ± 2.74	0.098	0.907
		One-year follow-up	–10.20 ± 1.79	–13.54 ± 1.52	–11.84 ± 0.96	0.992	0.372
		Change	0.23 ± 2.22	3.17 ± 1.39	1.27 ± 4.37	0.438	0.647
		*p*	0.739	0.183	0.295		
RV strain							
	GLSRV (%)						
		Baseline	–21.62 ± 4.28	–20.71 ± 5.28	–21.95 ± 6.29	0.383	0.683
		One-year follow-up	–21.77 ± 5.99^*^	–18.82 ± 5.25^‡^	–17.33 ± 3.93^†^	8.178	0.001
		Change	0.15 ± 2.39^*^	1.89 ± 2.31^‡^	4.62 ± 3.13^†^	3.882	0.025
		*p*	0.511	0.203	0.029		
	RVFWLS (%)						
		Baseline	–26.4 ± 6.05	–24.24 ± 7.07	–26.92 ± 8.18	0.066	0.936
		One-year follow-up	–26.43 ± 5.53^*^	–22.71 ± 4.52^‡^	–22.1 ± 5.92^†^	5.166	0.008
		Change	0.03 ± 1.52	1.53 ± 2.31^‡^	4.82 ± 3.13^†^	2.151	0.024
		*p*	0.939	0.353	0.025		
	LV-RV TPSD						
		Baseline	16.41 (8.81–42.5)	18.91 (9.5–40.7)	16.47 (9.2–43.9)	0.302	0.740
		One-year follow-up	12.28 (5.64–23.7)	25.34 (8.81–53.6)	44.8 (19.6–147.5)	9.123	0.001
		Change	4.13 ± 4.99^*^	6.42 ± 6.62^‡^	28.8 ± 7.74^†^	8.345	0.001
		*p*	0.001	0.001	0.001		

Abbreviations: LBBAP, left bundle branch area pacing; RV,right ventricular; GLSRV, global longitudinal strain of the right ventricle; RVSP, right ventricular septal pacing; RVAP, right ventricular apical pacing; STE, speckle tracking echocardiography; RA, right atrial; RVFWLS, RV free wall longitudinal strain; TPSD, time-to-peak standard deviation; LV-RV TPSD, left ventricle–right ventricle time-to-peak standard deviation. ^†^*p* < 0.05 RVAP vs. LBBAP, ^*^p < 0.05 LBBAP vs. RVSP, ^‡^*p* < 0.05 RVSP vs. RVAP.

### 3.3 TR Regurgitation

Mechanical dysynchrony may be related to the severity of regurgitation. At the 1-year follow-up, patients who had undergone RVSP or RVAP had more severe TR compared to baseline, but this change in both TRVCM (0.33 ± 0.23 cm with baseline vs. 0.34 ± 0.11 cm with follow-up, *p* = 0.467) and TR jet area/right atrial area (RAA) (13.46 ± 1.33% vs. 13.34 ± 3.39%, *p* = 0.667) was not apparent in patients with LBBAP. Also at the 1-year follow-up, significant differences in the TR jet area/RAA (13.34% ± 3.39% vs. 15.44% ± 2.99% vs. 15.42% ± 3.90%, respectively; *p* = 0.015) and TR VCM (0.34 ± 0.11 cm vs. 0.40 ± 0.10 cm vs. 0.41 ± 0.12 cm, respectively; *p* = 0.035) were observed between patients who received LBBAP and those who received RVSP and RVAP (Table 2).

### 3.4 RA Function

Among the groups, no statistically significant variation was identified in the structural or strain parameters of the RA, specifically the RA reservoir, conduit, and contraction strain, at either the initial assessment or over the 1-year follow-up period (Table 3).

### 3.5 Electrical and Mechanical Synchronization

As shown in Table 1, the QRS duration, which is regarded as a marker of electrical synchronization, was slightly increased in the LBBAP group (from 111.26 ± 24.07 ms to 114.45 ± 26.23 ms, *p* = 0.783), whereas QRS duration significantly increased in both RVSP (from 102.88 ± 18.78 ms to 133.5 ± 28.34 ms, *p* = 0.001) and RVAP (from 101.55 ± 16.09 ms to 157.29 ± 29.18 ms, *p* = 0.001). The LBBAP group showed a significant decrease in IVMD before and after pacemaker implantation (from 17.3 ± 26.5 to 8.6 ± 7.1 ms, *p* = 0.001), whereas IVMD increased significantly in both RVSP (from 17.6 ± 29.8 ms to 29.7 ± 9.9, *p* = 0.034) and RVAP (from 17.4 ± 28.4 to 44.7 ± 12.8, *p* = 0.001) (Table 2). Also, the LBBAP group showed a significant decrease in LV-RV TPSD during the 1-year follow-up [from 16.41 (8.81–42.5) to 12.28 (5.64–23.7), *p* = 0.001], while the LV-RV TPSD increased significantly during follow-up in both the RVSP group [from 18.91 (9.5–40.7) to 25.34 (8.81–53.6), *p* = 0.001] and RVAP group [from 16.47 (9.2–43.9) to 44.8 (19.6–147.5), *p* = 0.001] (Table 3).

## 4. Discussion

This cohort study evaluated the efficacy of LBBAP, RVSP, and RVAP in pacemaker-dependent patients, focusing mainly on the structure, function, and interventricular synchronization of the right heart from an echocardiographic view at the 1-year post-operative follow-up. The major findings are as follows: (1) LBBAP improves mechanical synchrony, in terms of both IVMD and LV-RV TPSD on STE, but RVAP and RVSP lead to mechanical desynchronization; (2) During the mid-term observation period, LBBAP has been observed to preserve RV function, as assessed by parameters including RVFAC, TAPSE, RVMPI, GLSRV, and RVFWLS. In contrast, RVAP and RVSP have been associated with a deterioration in these functional measures; and (3) LBBAP has not been found to exacerbate TR when compared with RVAP and RVSP.

As supported by our previous research [4, 15, 16], LBBAP has become a prominent physiological pacing technique as ample evidence has demonstrated that it can enhance LV function and intraventricular synchronization. Nevertheless, whether RV delay induced by incomplete RBBB patterns occurring after LBBAP affects right heart structure, function, and interventricular mechanical synchronization has not been comprehensively investigated. Additional research showed that LBBAP is an excellent indicator of fast and synchronous biventricular contraction [17], and a similar study reported that LBBAP is effective at improving mechanical synchronization based on the use of peak strain dispersion (PSD) and IVMD as parameters for assessing synchronization [18]. In the present study, we consistently confirmed that LBBAP led to significantly better functional outcomes compared with RVSP and RVAP in terms of QRS duration, IVMD, LV-RV TPSD even in the presence of a pacing pattern resembling RBBB. This phenomenon may be attributed to the fact that LBBAP initiates ventricular activation in close proximity to the physiological conduction system. The earliest activated segments correspond to the mid-basal interventricular septum, whereas the latest activation is observed in the LV lateral wall and RV free wall.

In pacemaker-dependent patients, structural and functional changes in the right heart after pacemaker implantation represent a topic of increasing concern. The RV and LV are interdependent due to their shared structures, including the interventricular septum, pericardial space, and myocardial fibers, which play a crucial role in their coordinated function. RV dysfunction may cause changes in the geometry of LV, resulting in impaired LV filling and a decrease in cardiac output [19]. Current protocols for echocardiographic quantification of RV function advise employing a variety of indices—including RVMPI, TAPSE, RVFAC, S’-wave, and others—to provide a comprehensive and exhaustive description of RV activity. To further improve the reliability of such findings, assessment of GLSRV and RVFWLS was conducted in our study using STE analysis. This approach is regarded as a new and promising technique that offers exceptional temporal resolution for the evaluation of RV systolic function [20]. Recent studies have demonstrated that assessment of peak global longitudinal RV strain, specifically omitting the interventricular septum, holds predictive significance in a range of clinical conditions, including heart failure [21], acute myocardial infarction [22], and RV failure after implantation of an LV assist device [23]. A previous study has also demonstrated that LBBP induces an excellent electrical and mechanical resynchronization and can lead to significant improvement in RV volumes and function [24]. Consistent with previous research [25, 26, 27], LBBAP more effectively preserved RV morphological parameters such as RVFAC, TAPSE, RVMPI, GLSRV, and RVFWLS compared with RVSP or RVAP in the present study. These findings suggest that LBBAP may be able to protect RV function and hemodynamics by maintaining LV function and ensuring interventricular and RV mechanical synchrony.

Permanent pacemakers have the potential to induce or exacerbate TR. TR deterioration was significantly associated with an increased risk of worsening congestive heart failure and decreased survival [28, 29]. Prior research established a correlation between pacemaker-induced TR and a multitude of variables, such as ventricular synchrony, the functionality of the LV and RV, the disruption to the tricuspid valve apparatus caused by the lead, and others [30]. Hu *et al*. [31] and Li *et al*. [32] demonstrated that the distance from the lead implanted site to the septal leaflet of the tricuspid annulus might be a major factor influencing TR deterioration after LBBAP implantation. The 2021 Chinese expert consensus is distinguished as the sole guideline advocating for the implementation of left bundle branch pacing (LBBP) on the right aspect of the interventricular septum (IVS), specifically at a distance of approximately 10 to 20 millimeters from the septal leaflet of the tricuspid valve. A previous similar study has shown that there were no significant deterioration in TR for the LBBAP group [27]. In the current study, after 1 year of observation, significant differences in the TR VCM and TR jet area/RAA were observed among groups treated with LBBAP, RVSP, and RVAP procedures. In addition to the aforementioned findings, the present study has observed that LBBAP does not exacerbate TR, whereas RVSP and RVAP have been associated with deleterious effects on TR. This may be because LBBAP involves the use of thinner, gentler electrode wires and promotes favorable intraventricular and interventricular synchrony. Interestingly, a study by Li *et al*. [32] suggested a comparable TR worsening risk in both LBBAP and RVSP in patients with LVEF >40%. The possible reasons for the differences in our conclusions may lie in the fact that our study population consisted of patients with a normal LVEF who were dependent on pacemakers (ventricular pacing (VP) >70%), and we used a quantitative method for assessing TR such as TR VCM and TR jet/RAA instead of comparing based on the severity grading of mild, moderate, and severe.

## 5. Limitations

The present study was a single-center, observational, prospective cohort study with a relatively limited sample size. To establish the safety and therapeutic advantages of LBBAP in comparison to RVSP or RVAP, extensive randomized controlled studies with larger sample sizes and extended follow-up periods are required.

## 6. Conclusions

Compared with RVSP or RVAP, LBBAP can maintain RV function and improve electrical and interventricular synchrony, with limited TR deterioration after a 1-year follow-up.

## Availability of Data and Materials

The datasets used and analyzed during the current study are available from the corresponding author YW on reasonable request.
